# Is signal intensity of late gadolinium enhancement a substitute for extracellular volume mapping in acute myocardial infarction?

**DOI:** 10.1186/1532-429X-17-S1-P156

**Published:** 2015-02-03

**Authors:** Pankaj Garg, Ananth Kidambi, David P Ripley, Laura E Dobson, Peter P Swoboda, Tarique A Musa, Adam K McDiarmid, Bara Erhayiem, John P Greenwood, Sven Plein

**Affiliations:** Multidisciplinary Cardiovascular Research Centre & Leeds Institute for Cardiovascular and Metabolic Medicine, University of Leeds, Leeds, UK

## Background

Extracellular volume (ECV) calculated from T1 maps can quantitatively characterize myocardial tissue, including the severity of tissue damage in myocardial infarction (MI) (1). An alternative approach for measuring the density of MI may be to calculate the relative signal intensity of LGE images.) A direct comparison of signal intensity of LGE and ECV in MI has not been carried out. We aimed to compare these two parameters in patients presenting with acute myocardial infarction.

## Methods

**Figure 1:**
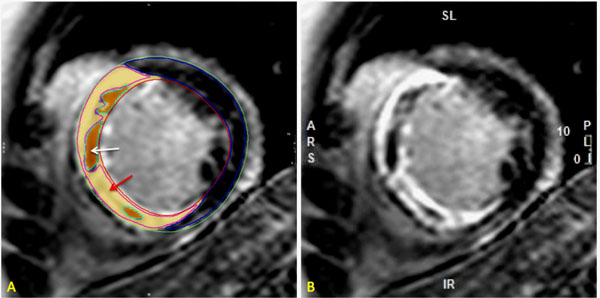
Semi-automated histogram-based thresholding method (Otsu method) was used to identify the infarct zone (red arrow) where a ROI was drawn to measure the SI. MVO (white arrow) is excluded from SI measurements.

Twenty-nine patients underwent CMR examination at 3T (Intera CV, Philips Healthcare, Best, The Netherlands) within 3 days following reperfused ST-elevation acute MI. Cine, pre and 15 minute post-contrast MOLLI (3(3s)3(3s)5) for ECV-mapping and LGE imaging (0.1 mmol/kg gadolinium DTPA) were performed. The slice with the largest area of LGE (core-of-the infarct) was chosen for further analysis. LGE in the infarct-zone was analysed with a semi-automated histogram-based thresholding method (Otsu method). Signal intensity was then recorded for a region of interest (ROI) in the infarct zone (excluding any microvascular obstruction; MVO) (Figure 1). ECV-mapping was carried out in the same slice as the infarct zone. ECV was also analysed for the remote myocardium. We computed relative ECV for the infarct zone using the formula = iECV-rECV)/rECV; where iECV is infarct-ECV and rECV is remote-myocardial-ECV.

## Results

**Figure 2:**
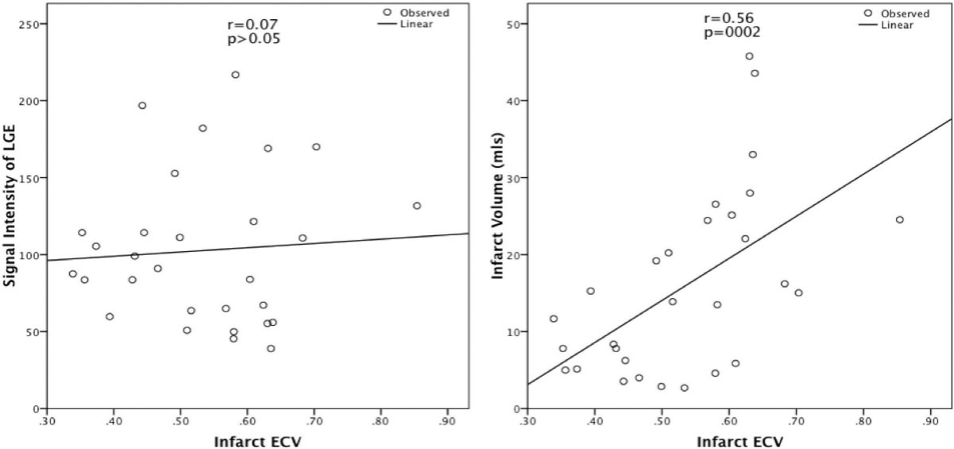
Scatter plot of correlation of infarct ECV to the signal intensity of LGE and to the infarct volume.

Patient characteristics of the 29 patients are as follows: mean age 57±28 years old; 82.8% males (n=24); 24% (n=7) had hypertension; 27.6% (n=8) had hypercholesterolemia; 55% (n=16) were smoker and 13.8% (n=4) had diabetes mellitus. CMR based parameters are as follows: ejection fraction (LVEF) 49±9%; infarct volume on LGE 15.9±11.8mls; iECV 53.4±12.1%; rECV 30±6%. There was significant difference in iECV to rECV (p<0.001). The volume of infarct was correlated to iECV (r=0.56; p=0.002) and also to relative ECV(r=0.45;p<0.05). There was no correlation of signal intensity of LGE in the infarct zone to the iECV (r=0.07; p=0.72) and to relative ECV(r=0.17;p=0.37) (Figure 2). The homogeneity of the signal intensity was also not related to iECV (r=0.14; p=0.45) and relative ECV (r= 0.18; p=0.35).

## Conclusions

Signal intensity of LGE in patients presenting with acute-MI is not correlated to the ECV in the infarct zone or to the relative ECV. ECV mapping is required to quantify the extent of tissue injury in acute MI.

## Funding

JPG and SP receive a research grant from Philips Healthcare. SP is funded by British Heart Foundation fellowship (FS/10/62/28409).
